# Irisin response to downhill running exercise in humans

**DOI:** 10.20463/jenb.2018.0011

**Published:** 2018-06-30

**Authors:** Yoshifumi Tsuchiya, Sahiro Mizuno, Kazushige Goto

**Affiliations:** 1 Graduate School of Sport and Health Science, Ritsumeikan University, Shiga Japan; 2 Research Fellowship for Young Scientists of the Japan Society for the Promotion of Science, Tokyo Japan; 3 Faculty of Sports and Health Science, Ritsumeikan University, Shiga Japan

**Keywords:** Irisin, downhill running, exercise-induced muscle damage, FNDC5, myoglobin, IL-6

## Abstract

**[Purpose]:**

To determine the effects of exercise-induced muscle damage, we examined irisin responses during level running (LR), with less muscle damage, and downhill running (DHR), with greater muscle damage under equivalent exercise duration and oxygen consumption (⩒O_2_) conditions.

**[Methods]:**

Fifteen healthy men (age: 21.6 ± 2.0 y, height: 170 ± 1.3 cm, weight: 64.8 ± 2.7 kg) were randomly assigned to either the LR group (n = 8) or the DHR group (n = 7). Subjects in the LR group performed treadmill running at 70% of maximum oxygen uptake (⩒O_2max_) for 30 min on a 0% gradient. In contrast, subjects in the DHR group performed the same exercise on a –10% gradient. Blood samples were collected before exercise, immediately after exercise, and 1, 3, and 24 h after exercise.

**[Results]:**

No significant interaction (group × time) or main effect of group or time was observed for changes in plasma irisin concentrations over time (P > 0.05). However, the area under the curve of plasma irisin concentrations during a 3-h post-exercise period was significantly greater in the DHR (239,197 ± 8,166 ng/mL) group than in the LR (92,293 ± 8,755 ng/ml) group (P < 0.05). The blood lactate, serum cortisol, myoglobin, and plasma interleukin-6 concentrations were significantly higher in the DHR group than in the LR group after exercise (P < 0.05 for all variables).

**[Conclusion]:**

DHR associated with marked muscle damage promoted a greater increase in exercise-induced irisin did LR after the same duration under identical VO2 conditions.

## INTRODUCTION

Myokines are physiologically active substances released from skeletal muscle^1^. Irisin is a relatively recently discovered myokine that enhances energy expenditure (EE) by increasing UCP1 expression in white and/or beige adipocytes^2,3^. Several studies have described the irisin response to a single bout of exercise^4-10^. However, the effect of endurance exercise on the irisin response remains unclear, although the mode of exercise may affect the response. The plasma irisin concentration increased significantly at 54 min during 90 min of running^11^, but not after 60 min of pedaling^12^. In contrast, a single bout of resistance exercise (6–8 exercises) significantly increased the irisin response^13,14^. Notably, resistance exercise caused a greater increase in irisin concentration than did the same duration of endurance exercise (pedaling), although the EE was much lower during resistance exercise^12^. Differences in the exercise-induced irisin response among various studies may be explained by the different exercise modalities, with varying degrees of eccentric contraction. During eccentric contraction, fast-twitch fibers are more prominently recruited than during concentric contraction^15-18^. An increase in exercise intensity promotes recruitment of fast-twitch fibers, leading to an augmented irisin response^19^.

Downhill running (DHR) especially highlights eccentric contraction in the quadriceps femoris compared with level running (LR), causing severe exercise-induced muscle damage^20-22^ and inflammation^23^ during the post-exercise period. These factors cause damage to neutrophils and monocytes due to exercise-induced oxidative stress^24^. Huh et al. showed that exercise-induced elevation of irisin was observed with a concomitant increase in creatine kinase (CK) (an indirect muscle damage marker)^25, 26^. However, it remains unclear whether the magnitude of exercise-induced muscle damage influences the irisin response. Therefore, we examined the irisin responses in 2 different types of running exercise: DHR and LR under similar EE conditions. We hypothesized that the irisin response would be augmented more after DHR than after LR.

## METHODS

### Subjects

Fifteen healthy, physically active men [mean age ± standard error (SE): 21.6 ± 2.0 y, height: 170 ± 1.3 cm, weight: 64.8 ± 2.7 kg, body mass index: 22.4 ± 0.8 kg/m^2^] participated in the present study. All participants had undergone several years of exercise training and exercised at least weekly. They were informed of the study purpose, experimental procedures, and possible risks of the study, and they provided written informed consent. All experimental procedures were approved by the Ethics Committee for Human Experiments at Ritsumeikan University (IRB-2014-004) and all complied with the Helsinki Declaration.

### Experimental setting

The subjects visited the laboratory twice during the experimental period. On the first day, maximum oxygen consumption (⩒O_2max_) during treadmill running was determined. On the second day (main experiment day), all subjects completed either a 30-min LR or a DHR protocol on a treadmill (Valiant Lode B. V., Groningen, the Netherlands). Exercise-induced irisin and metabolic responses were monitored during a 24-h post-exercise period.

**Fig. 1.**
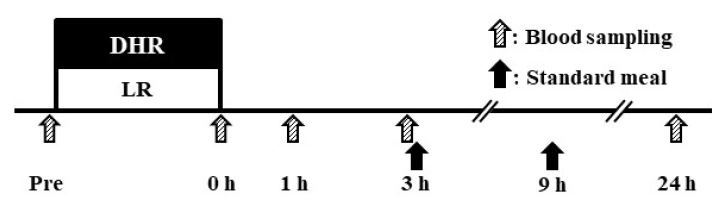
Time frame of main experiment.

Fifteen subjects were randomly divided into either LR (*n* = 8) or DHR (*n* = 7) groups to match the ⩒O_2_ level between the groups. In the LR group, subjects ran for 30 min on a treadmill with a 0% gradient, whereas subjects in the DHR group ran with a –10% gradient. The exercise intensity was set to achieve 70% of ⩒O_2max_ during incremental running on a treadmill (0% gradient). The running velocity in the DHR group was adjusted individually during the initial 5 min of exercise to achieve 70% of the ⩒O_2max_. The DHR protocol was based on those of previous studies [27, 28] and our pilot study. All subjects were instructed to refrain from caffeine and alcohol and strenuous physical activity for 3 days before the trial day. They were also asked to minimize physical activity for 24 h after the exercise. Prescribed meals were provided at 3 h and 9 h after exercise (Figure 1). All subjects were allowed to consume only water ad libitum on the day of the trial. The sleep duration was set from 22:00 to 07:00.

### ⩒O_2max_

Approximately 1 week prior to the main experiment, the ⩒O_2max_ was evaluated to determine running velocity. Respiratory gas samples during exercise were collected with breath-by-breath method using an automatic gas analyzer (AE300S Minato Medical Science, Osaka, Japan). All data were averaged every 30 s. The treadmill gradient during the ⩒O_2max_ test was set at 0%. The ⩒O_2max_ test commenced with running at 6.0, 8.0, and 10.0 km/h for 4 min each. The running speed was then increased by 2.0 km/h every 3 min to 12.0 km/h and 14.0 km/h. When treadmill running at 14.0 km/h was completed, the speed was progressively increased every minute by 0.6 km/h to exhaustion. Exhaustion was defined as 1) ⩒O_2max_ plateau; 2) a heart rate equal to the maximum age-predicted value (220 minus age); or, 3) a respiratory exchange ratio (RER) of 1.1. When a subject met at least 2 of these 3 criteria, the test was concluded.

### Respiratory gas sampling during exercise

On main experiment days, respiratory gas samples during 30 min (8:40-9:10) of treadmill running were evaluated with the breath-by-breath method using an automatic gas analyzer (AE300S Minato Medical Science, Osaka, Japan). Respiratory gas samples were used for continuous determination of ⩒O_2_, carbon dioxide output (⩒CO_2_), minute ventilation (⩒E), and the RER. All data were averaged every 30 s.

### Blood samples

The subjects presented at 8:00. First, a venous blood sample (i.e., pre-exercise sample) was collected after 30 min of rest. The subjects began prescribed exercise at 8:40. Additional blood samples were collected immediately after exercise (9:10), and at 1 h (10:10), 3 h (13:10), and 24 h (09:10) after exercise (Figure 1). The collected blood samples were centrifuged for 10 min (3,000 rpm, 4°C), and the serum and plasma samples were stored at −60°C prior to analysis. Blood samples were assessed immediately after collection with automated glucose (Freestyle; Nipro Co., Osaka, Japan) and lactate (Lactate Pro2 LT- 1730; Arkray Inc., Kyoto, Japan) analyzers. Serum cortisol, myoglobin, and CK concentrations were measured at a clinical laboratory (SRL Inc., Tokyo, Japan). Plasma interleukin (IL)-6 and irisin concentrations were determined using enzyme-linked immunosorbent assay (ELISA) kits [HS600B; R&D Systems, Minneapolis, MN, USA (IL-6); EK-067-52 (Lot 605767); Phoenix Pharmaceuticals, Inc., Darmstadt, Germany (irisin)]. All samples for ELISA were assayed in duplicate and the results were averaged. The intra-assay coefficients of variation were: 2.5% for serum cortisol, 2.4% for serum myoglobin, 2.3% for serum CK, 2.5% for plasma IL-6, and 4.3% for plasma irisin.

### Statistical analysis

All experimental data are shown as means ± SE. An unpaired t-test was used to explore the significance of differences between groups for respiratory gas data and the area under the curve (AUC) of plasma irisin concentrations. Moreover, two-way (group × time) repeated analysis of variance (ANOVA) was applied to identify significant interactions and main effects. When a significant interaction and main effect were observed, the data were subjected to post-hoc analysis (Tukey-Kramer test). *P* < 0.05 was considered to reflect significance. Cohen’s d (for the unpaired t-test) and the η^2^ value (for 2-way ANOVA) were calculated, as appropriate, to evaluate effect sizes.

## RESULTS

### Respiratory gas variables during exercise

Table 1 presents the respiratory gas variables during the 30 min of exercise in each group. The average ⩒O_2_, ⩒CO_2_, ⩒E, and energy expenditure (EE) during exercise did not differ significantly between the groups (*P* = 0.35, *d* = 0.50 for ⩒O_2_; *P* = 0.60, *d* = 0.28 for ⩒CO_2_; *P* = 0.62, *d* = 0.26 for ⩒E; and *P* = 0.39, *d* = 0.46 for EE, respectively). However, the average RER during exercise was significantly higher in the DHR group (*P* = 0.03, *d* = 1.23).

**Table 1.** Respiratory gas variables during exercise in each group.

Variables	LR(n=8)	DHR(n=7)	*P* Value
⩒O_2_(mL/min)	2,443 ± 192	2,205 ± 139	0.35
⩒CO_2_(mL/min)	2,268 ± 188	2,143 ± 129	0.60
⩒E(mL/min)	66.3 ± 7.5	71.4 ± 6.5	0.62
RER	0.93 ± 0.01	0.97 ± 0.02^†^	0.03
EE(kcal)	361 ± 29	329 ± 20	0.39

Values are means ± SE. ^†^: P < 0.05 vs. LR group.

### Blood variables

**Fig. 2.**
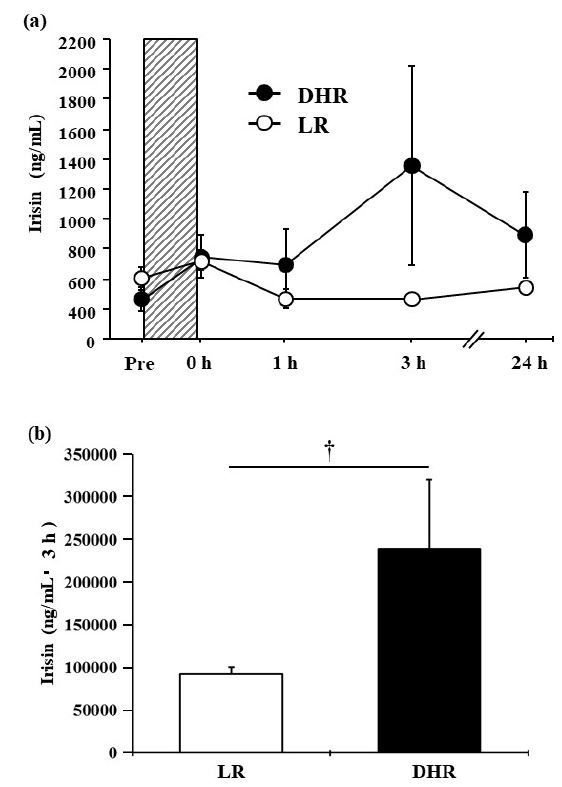
(a) Plasma irisin concentrations before and after each exercise. Shaded box: exercise period. (b) Area under the curve (AUC) of plasma irisin concentration during the 3-h post-exercise period. Values are means ± SE. ^†^: *P* < 0.05.

Figure 2 a presents the time-course changes in plasma irisin concentrations. No significant interaction (group × time, *P* = 0.159, η^2^ = 0.07) or main effect for group (*P* = 0.25, η^2^ = 0.04) and time (*P* = 0.30, η^2^ = 0.04) was evident. In the DHR group, and the plasma irisin concentration increased approximately 3-fold by 3 h after exercise (before: 466.1 ± 78.8 ng/ml; 3-h: 1,358.0 ± 666.7 ng/mL), but the difference did not reach significance (*P* = 0.20, *d* = 0.74). When the exercise-induced irisin responses over the 3-h post-exercise period were compared, the AUC was significantly greater in the DHR group than in the LR group (*P* = 0.04, *d* = 0.99; Figure 2b).

**Fig. 3.**
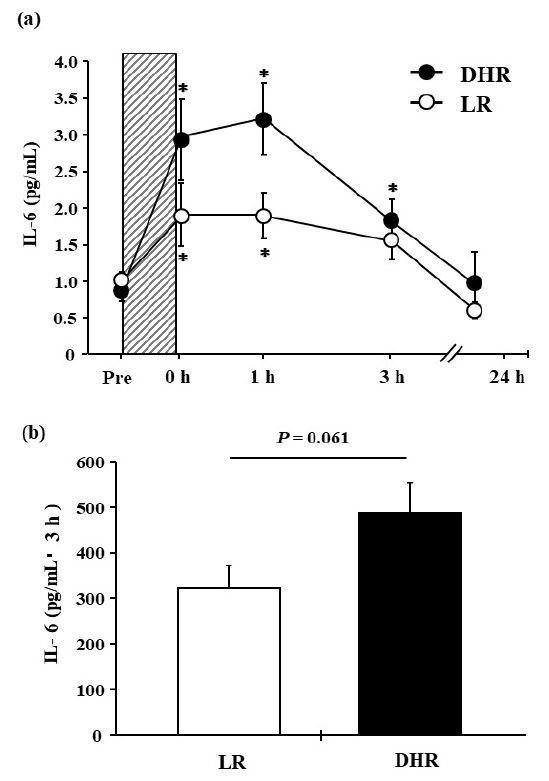
(a) Plasma IL-6 concentrations before and after each exercise. Shaded box: exercise period. (b) AUC of plasma IL-6 concentration during the 3-h post-exercise period. Values are means ± SE. ^*^: *P* < 0.05 vs. Pre.

As shown in Figure 3a, there was no significant interaction (group × time; *P* = 0.122, η^2^ = 0.06) or main effect for group (*P* = 0.105, η^2^ = 0.10) for the plasma IL-6 concentration. Plasma IL-6 concentrations increased significantly immediately (0 h) and 1 h after exercise in both groups (main effect for time; *P* = 0.001, η^2^ = 0.37). Moreover, the DHR group showed a significant increase at 3 h after exercise (*P* = 0.002, *d* =1.51). The AUC over the 3-h post-exercise period for plasma IL-6 tended to be greater in the DHR group than in the LR group (*P* = 0.061, *d* = 1.06, Figure 3b).

Table 2 shows the time-course changes in blood variables. No significant interaction (group × time) was evident for blood glucose (*P* = 0.07, *η*^2^ = 0.09), lactate (*P* = 0.16, η^2^ = 0.06), or serum CK concentrations (*P* = 0.22, η^2^ = 0.1). There was no significant main effect for group for blood glucose (*P* = 0.20, η^2^ = 0.01), serum cortisol (*P* = 0.06, η^2^ = 0.14), or CK concentrations (*P* = 0.22, η^2^ = 0.15). The blood glucose concentration exhibited a significant main effect for time (*P* < 0.001, η^2^ = 0.54), and there was a significant increase in the DHR group (*P* < 0.001, *d* =1.19), but not in the LR group. The blood lactate concentration exhibited a significant main effect for time (*P* < 0.05, η^2^ = 0.44) and a tendency toward significant main effect for group (*P* = 0.06, η^2^ = 0.04). Blood lactate concentration increased significantly immediately after exercise in both groups (LR: *P* = 0.002, *d* =1.19; DHR: *P* < 0.001, *d* =1.62). Furthermore, the blood lactate concentration immediately after exercise was significantly higher in the DHR group than in the LR group (*P* < 0.001, *d* =0.85). The serum cortisol concentration exhibited a significant interaction (group × time, *P* = 0.005, η^2^ = 0.06) and a main effect for time (*P* < 0.001, η^2^ = 0.21). The DHR group revealed significantly higher values both immediately and 1 h after exercise (0 h: *P* < 0.001, *d* =1.53 and 1 h: *P* < 0.001, *d* =1.43). The serum myoglobin concentration exhibited a significant interaction (group × time, *P* < 0.003, η^2^ = 0.22) and main effects for group (*P* < 0.001, η^2^ = 0.18) and time (*P* = 0.003, η^2^ = 0.26). In the DHR group, the myoglobin concentration was significantly higher than in the LR group immediately (*P* = 0.01, *d* = 1.28), and 1 (*P* < 0.001, *d* = 2.28) and 3 h after exercise (*P* < 0.001, *d* = 1.82). The serum CK concentration did not exhibit a main effect for time (*P* = 0.12, η^2^ = 0.17).

**Table 2.** Time-course changes in blood variables in each group.

Variables		Pre	0 h	1 h	3 h	24 h
Glucose (mg/dL)	LR	91 ± 1	108 ± 10	86 ± 2	86 ± 2	90 ± 1
	DHR	87 ± 2	133 ± 8 ^*^	81 ± 3	87 ± 2	91 ± 2
Lactate (mmol/L)	LR	1.5 ± 0.2	3.5 ± 0.8 ^*^	1.5 ± 0.1	1.5 ± 0.2	1.4 ± 0.1
	DHR	1.8 ± 0.2	6.1 ± 1.4 ^*†^	2.1 ± 0.1	1.6 ± 0.1	1.6 ± 0.1
Cortisol (μg/dL)	LR	17.2 ± 2.2	18.1 ± 2.4	16.4 ± 2.5	11.8 ± 1.9 ^*^	17.5 ± 1.5
	DHR	21.1 ± 3.0	28.0 ± 2.3 ^†^	26.7 ± 2.8 ^†^	13.9 ± 1.5 ^*^	19.6 ± 2.5
Myoglobin (ng/mL)	LR	39.3 ± 5.6	63.3 ± 9.0	87.3 ± 9.9	79.1 ± 10.1	40.9 ± 3.3
	DHR	60.0 ± 29.7	207.7 ± 62.1^*†^	564.0 ± 115.9 ^*†^	483.7 ± 109.5 ^*†^	68.2 ± 23.4
CK (U/L)	LR	220 ± 55	241 ± 62	275 ± 54	261 ± 53	246 ± 30
	DHR	606 ± 467	712 ± 514	751 ± 525	877 ± 505	1070 ± 307

Values are means ± SE.

^*^: P < 0.05 vs. Pre.

^†^: P < 0.05 vs. LR group.

## DISCUSSION

This is the first study to explore the effects of exercise- induced muscle damage on the irisin response. As expected, exercise-induced elevation of serum myoglobin (an indirect muscle damage marker) and plasma IL-6 (an inflammatory cytokine) were significantly greater in the DHR group than in the LR group, although EE during 30 min of exercise was not significantly different. Consequently, the plasma irisin concentration increased approximately 3-fold 3 h after completion of DHR (Pre: 466.1 ± 78.8 ng/mL, 3 h after exercise: 1,358.0 ± 666.7 ng/mL). Additionally, the exercise-induced irisin response over 3 h after DHR was significantly greater than that after LR, suggesting that the augmented irisin response may be associated with magnitude of muscle damage.

Exercise intensity is a primary factor in irisin production^19,26^, and increased recruitment of fast twitch fibers may play an important role in the irisin response. During DHR, eccentric muscle contraction (which preferentially recruits fast twitch fibers) is highlighted during the landing phase^15-18^. Furthermore, running velocity was significantly higher in the DHR group (14.0 ± 0.2 km/h) than in the LR group (10.8 ± 0.3 km/h), to match EE during exercise. We previously showed that higher-velocity running was associated with a greater irisin response than lower-velocity running under matched EE conditions^19^. Therefore, enhanced irisin response (revealed by the AUC over 3 h) after DHR might be, at least in part, explained by higher running velocity with increased recruitment of fast twitch fibers.

We sought to identify associations between the levels of exercise-induced muscle damage markers (myoglobin and CK) in blood and the irisin response. Several studies suggested a relationship between these 2 factors in both humans^26^ and rats^29^. However, the above studies involved limitations, including low frequencies of blood collection. In the present study, we monitored the time course of changes in the levels of plasma irisin and muscle damage markers over a 24-h post-exercise period. The serum myoglobin level was markedly higher in the DHR group than in the LR group, indicating that exercise-induced muscle damage was profound in the DHR group. Moreover, exercise- induced plasma IL-6 elevation was profound in the DHR group (until 3 h post-exercise). The AUC value over a 3-h post-exercise period tended to be greater in the DHR group than in the LR group (*P* = 0.06). Similarly, Vyver et al. showed that both serum myoglobin and IL-6 concentrations increased concomitantly after completion of DHR^30^. Therefore, significantly higher plasma IL-6 concentration during the post-exercise period in the DHR group may be related to proinflammatory action following ultrastructural muscle damage. The exercise-induced elevation of lactate was also greater in the DHR group than in the LR group, suggesting that DHR promoted muscle glycogen utilization. Previous studies suggested that a high level of IL-6 was seen with low glycogen levels, and that the lower glycogen level induces PGC-1α (an upstream factor in irisin production) gene expression^31-33^. Additionally, Huh et al. found that the mRNA levels of PGC-1α (an upstream factor in irisin production) and FNDC5 (the precursor of irisin) were increased by IL-6 addition in vitro^13^. Thus, further reduction of muscle glycogen levels caused by DHR may promote the PGC-1α-FNDC5-irisin axis via IL-6 production. However, it is difficult to clarify completely the association between IL-6 and irisin, since IL-6 has multiple sources (e.g., leucocytes, myocytes) and actions (pro- and anti-inflammatory). Taken together, the present data suggest that the exercise-induced irisin response may be associated with muscle damage (reflected by the elevation in myoglobin concentrations) and/or inflammation (reflected by the elevation in IL-6 concentrations).

In the present study, post-exercise increases in irisin levels were modest compared to those of previous studies^11^. Although the intensity (70% of VO_2max_) and duration (30 min) during DHR exercise appeared to be sufficient to stimulate irisin production, strenuous muscle contraction can trigger myokine proteolysis^34^. Therefore, it is possible that excessive muscle damage caused by DHR attenuated the transient irisin response.

Several limitations should be noted when interpreting the results. First, we did not use a crossover design, because we attempted to avoid “repeated bout effects” for exercise-induced muscle damage^35^. Thus, we divided the 15 subjects into DHR and LR groups, and inter-individual variation in the irisin response should be considered. Second, our results may be specific to healthy subjects. Indeed, obese subjects and patients with type 2 diabetes exhibited irisin responses different from those of healthy individuals^34^. Third, the existence of irisin and its role in humans is controversial^37-39^. However, Jedrychowski et al. used quantitative mass spectrometry to precisely measure plasma irisin levels in healthy humans in both the resting and post-exercise state^40^. Furthermore, the ELISA that we used in the present study was the most commonly employed in previous studies^25,36,41-44^.

From a practical viewpoint, our findings will aid in the design of exercises that efficiently reduce the risk of metabolic disorder and associated disease. We speculate that exercise-induced irisin secretion would be influenced by muscle damage and inflammation. A future study should address the physiological implications of transient increases in irisin levels on the resting metabolic rate, insulin sensitivity, and body composition in various populations.

In conclusion, we found that the exercise-induced irisin response over the 3 h period after DHR was significantly greater than that after LR, although the EE was similar.
